# The impact of a gender transformative school-based suicide prevention workshop in Australia: adolescent boys’ perspectives

**DOI:** 10.1093/heapro/daaf160

**Published:** 2025-09-29

**Authors:** Dana Meads, Katherine A Lawrence, Glenn Melvin, Anna Clark, Patty Chondros, Kylie King

**Affiliations:** School of Psychological Sciences, Monash University, Boonwurrung Country, Clayton, VIC, 3800, Australia; School of Psychological Sciences, Monash University, Boonwurrung Country, Clayton, VIC, 3800, Australia; School of Psychological Sciences, Deakin University, Wurundjeri Woi-wurrung Country, Burwood, VIC, 3125, Australia; School of Psychological Sciences, Monash University, Boonwurrung Country, Clayton, VIC, 3800, Australia; Department of General Practice and Primary Health Care, The University of Melbourne, Wurundjeri Woi-wurrung Country, Parkville, VIC, 3010, Australia; School of Psychological Sciences, Monash University, Boonwurrung Country, Clayton, VIC, 3800, Australia

**Keywords:** gender transformative, help-seeking, male, youth, school-interventions, suicide prevention, masculinities

## Abstract

Elevated suicide rates among adolescent boys in Australia reflect a critical need to address restrictive masculine norms that hinder help-seeking. Tomorrow Man’s ‘Breaking the Man Code’ workshop—a school-based gender transformative programme—aims to increase authentic self-expression and meaningful dialogue among adolescent boys. Evidence of the impacts of gender transformative programmes is limited. Within a cluster randomized trial, 183 boys (Years 10 upwards) from 11 Australian schools completed open-ended survey questions about their experiences of the ‘Breaking the Man Code’ workshop and its perceived impact on help-seeking with friends and family. Data were analysed using reflexive thematic analysis. Three themes were developed: (i) the workshop normalized struggle and strengthened connection, creating a safe space for self-expression and peer connection; (ii) defining the ‘man code’ postworkshop: many participants expressed desires for increased self-expression and to support others; and (iii) talking openly with friends and family postworkshop. Greater relational impacts were described with friends, including increased compassion, trust, connection, and use of active listening skills. Perspectives within themes varied: while many discussed positive attitudinal and behavioural change, others reported no changes. The lack of reported change was largely attributed to pre-existing open communication practices; others gave no explanation; some noted difficulties implementing changes. Future research should further explore varied impacts among diverse boys. Analysis suggests that many adolescent boys in this sample desired and perceived that they could shift towards a positive masculinity characterized by deeper social connections, providing examples of this change within their relationships with friends and family postworkshop.

Contribution to Health Promotion Statement

- This research evaluates the impacts of a school-based suicide prevention programme that aims to reframe rigid masculine norms to increase help-seeking among adolescent boys.
- Findings suggest that many boys felt that the programme created a safe space for connection and encouraged increased self-expression and talking openly with friends and family.
- Qualitative methods offer crucial insights in understanding impacts of complex interventions addressing socially embedded constructs like masculine norms.
- Ongoing, community-wide support is needed to promote sustained changes.
- Future research should further explore differential impacts to tailor programmes for diverse groups of adolescent boys.

## BACKGROUND

In Australia, there are a range of intersecting populations among young people that are at elevated risk of suicide, including Aboriginal and Torres Strait Islanders, LGBTQIA+ and asylum seeker and refugee youth (Australian Institute of Health and Welfare [AIHW] 2025). Within this context, the suicide rate in the 15–24-year-old age group is almost three times higher for young men than it is for young women, reflecting a global trend (Australian Bureau of Statistics 2023; World Health Organisation [WHO] 2019). This disparity is influenced by reduced help-seeking among adolescent and young males, which is known to be strongly underpinned by masculine norms (Rasmussen *et al*. 2018, Sheikh *et al*. 2025). Masculine norms are defined as socially endorsed expectations of men and boys’ behaviours and expressions (Connell 2005). While masculine norms can have both positive and negative impacts on wellbeing (Iwamoto *et al*. 2018), rigid adherence to traditional norms encouraging self-reliance, stoicism, and restricted emotionality have been shown to actively inhibit help-seeking in situations of distress (Levant *et al*. 2009, Seidler *et al*. 2016, Clark *et al*. 2020).

Adolescent boys can interpret help-seeking as a sign of weakness, which can threaten their masculine identity status—an identity which prioritizes strength (Cleary 2012). This can create an internal conflict where essential help-seeking behaviours (i.e. reliance on others, vulnerability, flexible expression of emotions, and discussion of personal problems) can evoke feelings of shame and stigma (Rice *et al*. 2018a; Sheikh *et al*. 2025). Feelings of shame and stigma can lead to adolescent boys internalizing the belief that wanting to seek help means that they are weak or represents a personal deficit (Cole and Ingram 2020). This combination of shame, stigma, and reluctance to seek help may escalate suicide risk when adolescent boys’ experience turmoil or hardship. Consistent with this, higher conformity to self-reliance has been associated with suicidal thinking among young men aged 15–20 years (King *et al*. 2020). Qualitative studies emphasize the role of shame and the need to display self-reliance in accord with in-group masculine behaviour as potent barriers of help-seeking for adolescent boys, with young men aged 18–24 years reporting that they had felt compelled to project an image of strength and toughness throughout their secondary school years (Lynch *et al*. 2018; Rice *et al*. 2018b). This projection of toughness reflects an adherence to a masculine group identity that valorizes inflexible stoicism and self-reliance, which adolescent boys can be disinclined to compromise through help-seeking.

During adolescence, family and peer relationships are key socializing mechanisms of masculine norms, with boys aged 12–15 years experiencing pressure from both parents and peers to conform to traditional gender-typed behaviours (Chu *et al*. 2005 ; Jackson *et al*. 2021) in order to demonstrate their difference from girls (Galambos *et al*. 1990, Connell 2005 ). Although masculinity is an evolving heterogeneous construct, conformity to traditional norms, such as stoicism and self-reliance, is cited to be strongest during mid-to-late adolescence (aged 15–18 years) (Herreen *et al*. 2021). Young men are also known to overestimate their peers’ and societal endorsement of traditional masculine norms, which may intensify their perceived pressure to conform (Flood and The Men’s Project 2020). For adolescent boys, peer ‘policing’ may add to this dynamic, where boys use derogatory misogynistic or homophobic insults to prevent others from acting outside of traditional expectations, such as disdainfully categorizing particular behaviours as ‘feminine’ (Reigeluth and Addis 2016). More recently, King and colleagues (2020) found that higher adherence to heterosexuality norms was associated with decreased suicidal thinking among adolescent boys, suggesting that traditional masculine expectations remain influential and continue to create social sanctions for boys who diverge from dominant norms. Overall, this heightened conformity among adolescent boys is concerning as masculine norms that position men as rigidly strong, invulnerable, and stoic are juxtaposed with seeking help and remaining connected to peers and family for social and emotional support, creating a complex and potentially harmful dynamic for adolescent boys.

However, adolescence is also a critical period of identity exploration in which boys have the potential to actively challenge masculine norms and reciprocally shape the dynamics of their close relationships, particularly with peers and parents (Rogers and Way 2016, Rogers *et al*. 2021, Nielson *et al*. 2022). Adolescence, therefore, offers a pivotal time point for interventions that aim to shift harmful aspects of masculine norms and promote positive health behaviours, such as help-seeking, among young men.

### Upstream school-based gender transformative interventions

Early interventions that target young males and aim to relax rigid adherence to norms of self-reliance, stoicism, and restricted emotionality—and conversely, increase flexible expression of emotion and the open discussion of personal problems, thereby encouraging help-seeking—have been a longstanding goal of suicide prevention efforts (Canetto and Sakinofsky 1998). Schools, as primary sites of gender norm socialization, hold unique potential to shift gender-related social norms and help-seeking behaviours within the everyday social contexts of adolescent boys (Patton *et al*. 2000, Martino and Meyenn 2001). Such interventions may ultimately reduce the suicide risk among adolescent boys by reducing self-reliance, normalizing vulnerability, and increasing help-seeking (Rasmussen *et al*. 2018; Sheikh *et al*. 2025). Recently, the number of school-based programmes that seek to reframe masculine norms and increase flexible self-expression among adolescent boys has increased (Gwyther *et al*. 2019, Calear *et al*. 2021). These interventions are categorized as ‘gender transformative’, a term that is defined by the World Health Organisation as programmes that aim to critically evaluate and ultimately change harmful or maladaptive gender roles to improve gender equity in health outcomes and relationships (WHO 2007). This increase in school-based gender transformative programmes accords with the recent moves towards ‘healthy’ or ‘positive’ masculinity ideologies (Di Bianca and Mahalik 2022). Wilson and colleagues’ (2022) discuss how a positive masculinity might apply in school-based settings. The authors emphasize the importance of adolescent boys forming masculine identities that are centred on authentic connectedness to self and others, and intrinsic motivation to engage effectively with society, rather than relying on social pressures. These aspirations directly align with the core objectives of upstream gender transformative programmes that aim to challenge restrictive masculine norms and cultivate flexible self-expression among adolescent boys.

Despite the increasing prevalence of school-based gender transformative programmes, there is a scarcity of research that evaluates their effectiveness in improving mental health or suicide related outcomes by explicitly addressing masculine norms (Gwyther *et al*. 2019), highlighting a critical need for evaluation to build an evidence-base and identify best practices. Emerging evidence suggests that gender transformative school-based interventions utilizing role modelling and personal narratives may increase adolescent boys’ help-seeking intentions from friends (Calear *et al*. 2021). However, attitudinal shifts may not translate to behavioural change with focus group data indicating that boys can find it hard to continue talking openly with their friends following programmes (Elliott *et al*. 2022). Further research is needed to examine impacts and to understand the processes by which school-based gender transformative interventions effect change for adolescent boys.

### The ‘Breaking the Man Code’ workshop

The ‘Breaking the Man Code’ workshop is an upstream standardized public health intervention that aims to reduce male suicide risk by disrupting rigid masculine norms and cultivating protective factors, including enhanced self-expression, positive help-seeking attitudes, and encouraging deeper conversations that go ‘beyond the banter’ (www.tomorrowman.com.au). These factors directly counter rigid self-reliance and a reluctance to seek help, which, as discussed, are linked to increased suicide risk in boys and young men (King *et al*. 2020; Sheikh *et al*. 2025). The workshop frames the ability to talk about personal problems, express emotion, and demonstrate vulnerability as a skill; by practicing having deeper conversations boys can build ‘emotional muscle’, which may increase their future confidence to seek help when needed. The ‘Breaking the Man Code’ workshop runs for two hours and is delivered by facilitators from Tomorrow Man—an Australian social enterprise that conducts workshops for boys and men in schools, sporting clubs, workplaces, and communities. The workshop is implemented in secondary schools throughout Australia for male-identifying students in Year 10 and above. Approximately 100 adolescent boys typically participate per school, distributed across three groups of 30–35 students.

The workshop employs a participant-centred, experiential methodology, utilizing activities that encourage reflection on the ‘man code’ (i.e. collectively understood social rules for men, such as ‘boys don’t cry’) and relevant men’s mental health and suicide risk statistics within their personal lives. Peer leadership and collaborative learning are central to the approach, with a focus on creating space for students to decide to voluntarily share relevant personal narratives with their peers. Male role models facilitate the workshop, incorporating diverse verbal and nonverbal activities. For example, the ‘Step to the Line’ activity where students stand in two facing rows and physically step forward in response to statements that resonate with their personal experiences (e.g. having cried recently). These experiences, combined with activities where students practice active listening and reflection skills, aim to promote meaningful communication among adolescent boys. The core objective is to shift participants from rigid adherence to stoicism and self-reliance towards a more flexible expression of manhood that includes the ability to both be vulnerable and offer support, thereby facilitating a gender transformative process.

### The present study

This study adopts a relational focus by seeking to understand the impacts of the ‘Breaking the Man Code’ workshop on adolescent boys’ help-seeking behaviours within their close relationships with friends and family, recognizing the complex interplay between these key influences and individual agency in shaping adolescent masculinities and behaviour (Rogers *et al*. 2021). Consistent with calls to integrate adolescent boys’ voices and experiences into the evaluation of interventions targeting male youth (Rickwood *et al*. 2007, Burke *et al*. 2022), this research prioritizes boys’ perspectives. Exploring boys’ perceptions of the workshop’s impact within their relationships with friends and family is crucial for evaluating gender transformative interventions that aim to encourage vulnerability and self-expression, which may challenge existing relational dynamics.

As such, the current study aims to understand (i) participant experiences of the ‘Breaking the Man Code’ workshop, and (ii) participant perspectives of the workshop’s impact on their help-seeking about personal problems with friends and family.

## MATERIALS AND METHODS

The data presented in this study were gathered as part of a randomized controlled trial that sought to determine the impact of the ‘Breaking the Man Code’ workshops on participants. This article reports on the qualitative open-ended survey responses from participants allocated to the intervention group. Further details on the trial results and protocol are reported elsewhere (King *et al*. 2022; King *et al*. submitted).

Ethical approval was granted from Monash University’s Human Research Committee on 20 February 2020 (ID 22796). Recruitment targeted three Australian states where Tomorrow Man was most active: New South Wales, Victoria, and Western Australia. Department of Education approvals were granted in New South Wales (23/09/20) and Victorian (30/06/22) Departments of Education and Melbourne Archdiocese Catholic Schools (02/07/21). Department of Education approval was not granted in Western Australia. As such, independent schools in New South Wales, Victoria, and Western Australia, and state schools in New South Wales and Victoria, were eligible to participate. The trial was prospectively registered with ANZCTR on 2 September 2020 (ACTRN12620001134910, U1111-1253-6472).

### Delivery of the ‘Breaking the Man Code’ workshop

Tomorrow Man facilitators are sensitive to the unique environment of each workshop. Before running the workshop, facilitators are briefed about the context of the school and dynamics of students. School staff are not involved in the delivery of the workshop but can be present. School wellbeing staff are available at schools during and following the workshop to support students that may experience distress.

### Sample and recruitment

School participation in the study was authorized by the principals of participating schools. Schools were eligible for inclusion in the study if they: (i) requested a ‘Breaking the Man Code’ workshop for their male students in Year 10, 11, or 12 during 2021, 2022, or the first half of 2023; (ii) could accommodate the workshop within the designated intervention or waitlist period, as assigned by the researchers; and (iii) could disseminate study information to parents and provide two class times, spaced four to six weeks apart, for students to complete the baseline and follow-up questionnaires.

Student participants were eligible for inclusion if they: (i) were enrolled in Years 10, 11, or 12 at a participating school; (ii) were registered to participate in the ‘Breaking the Man Code’ workshop; (iii) had parental consent to participate in the study or were not actively opted out by their parent (opt-out consent procedures were implemented in Western Australia and New South Wales); and (iv) provided their own assent or consent to participate in the study.

There were no exclusion criteria. All students enrolled in a ‘Breaking the Man Code’ workshop at participating schools were eligible for inclusion.

### Design

This study is a post-intervention open-ended qualitative survey of students following participation in a workshop. Participants in the intervention group of a trial completed a baseline questionnaire battery within two weeks before the workshop and a follow-up questionnaire battery two to four weeks following the workshop. Both questionnaires measured outcomes for the trial (reported in trial paper) (King *et al*. submitted). The baseline questionnaire included demographic questions, while the follow-up questionnaire concluded with the open-ended survey questions.

### Data collection

Data were collected between 21 October 2020 and 4 April 2023. The baseline and follow-up survey each took approximately 20 minutes to complete. Students with consent to participate in the workshops and the study were sent online links to complete baseline and follow-up surveys during an allocated time at school together with other study participants. The lead researcher of this study (D.M.) extracted and analysed the open-ended survey questions of the follow-up survey and corresponding demographic data from baseline.

### Materials

The baseline questionnaire collected participant demographic information (including age, gender, sexual orientation, and cultural information) and school characteristics (including location and education type), as presented in the results section. Open-ended inquiries were designed by the research team to encourage a nuanced consideration of participants’ experiences (Yardley 2000). Participants were asked about their perceptions of the workshop (i.e. what parts did you like least/most) and the impact of the workshop on their ideas about the sort of man that they would like to be, and how they talk to family and friends about ‘personal stuff’. Given the need for brevity, the use of the word family was deliberately broad to acknowledge a variety of parental and carer relationships and allow participants to ascribe their own meaning. Students could answer or skip the questions. See Table 1 for survey questions.

**Table 1.** Survey questions.

Question no.	Questions
1	What parts did you like most about the workshop?
2	What parts did you like least about the workshop?
3	Did the workshop change your ideas about the sort of man you’d like to be? How did they change?
4	Did the workshop change the way you talk with your mates about personal stuff? How do you do it differently now?
5	Did the workshop change the way you talk with your family about personal stuff? How do you do it differently now?
6	How do you think your family would respond if you showed that you were sad or upset about a personal problem?
7	Is there anything else you want to tell us about the workshop?

### Data analysis

The socio-demographic descriptive statistics for the quantitative data were summarized using Microsoft Excel. These pre-workshop quantitative data were linked with the postworkshop qualitative data using a unique session key identifier. Qualitative data were managed using NVivo 14 (Lumivero). We undertook Braun and Clarke’s (2022) six step reflexive thematic analysis (TA) to develop themes across participant responses. Braun and colleagues (2021) emphasize that online surveys can gather rich qualitative data from large, geographically diverse samples, making them a valuable tool for capturing a wide range of perspectives and exploring emerging research topics. Moreover, the anonymous digital design can encourage candid responses and minimize power differentials, encouraging participation from adolescent boys who might otherwise hesitate to engage in research (Edwards and Holland 2020, McCarthy *et al*. 2023). The use of an online survey was therefore deemed suitable for the current study and notably for its capacity to allow patterns across the data to be developed (Braun *et al*. 2021). Responses to all survey items were pooled and analysed together. An inductive approach that incorporated a critical realist lens was employed by the coding researcher (D.M.) where analysis was primarily based on the content of participant responses, while also considering the coding researcher’s scholarly understanding of masculinity, gender, and developmental theories that may underpin participant narratives (Madill *et al*. 2000, Braun and Clarke 2006). The first author (D.M.) was the sole coder—in accord with recommended practices for this type of analysis (Braun and Clarke 2022)—and generated initial themes (constellations of shared meaning anchored by a core concept) and subthemes (distinct aspects within a theme) (Braun and Clarke 2006). To ensure ongoing reflexivity and critical engagement with the data and analysis, the themes were discussed with all authors. These discussions served to broaden and enrich the thematic analysis. As such, the themes were iteratively developed, reviewed, and refined by all authors, led by the first author.

We recognize the potential influence of the first author’s lived experiences on our research findings (Elliott *et al*. 1999; Yardley 2000). The first author, an adult woman with lived experience of male suicide, worked as a provisional psychologist with adolescent boys and adult men in settings characterized by a high risk of suicidality, suicide ideation, and self-harm during the analysis and writing of this research article. To critically examine the inherent subjectivity of the research process, the research team engaged in reflexive discussions, actively considering and challenging the perspectives and assumptions that both the lead researcher and the team may have brought to the analysis. This included acknowledging the belief that help-seeking (i.e. which includes flexible expression of emotion and vulnerabilities) is likely beneficial for adolescent boys and may serve as a protective factor against suicide. This perspective was balanced with the belief that the benefits of help-seeking may be contingent upon the availability and accessibility of support within individual and community social networks.

The results and discussion of reflexive analysis findings may be reported either together or separately (Braun and Clarke 2022). This study will present these components separately.

## RESULTS

This study reports on the 183 follow-up respondents within the intervention group of a trial who completed at least one qualitative open-ended question (mean of 15 years old; range: 14–17 years). This sample represents 56.3% of the 325 participants that completed both baseline and the follow-up questionnaire battery and 18.6% of the 985 participants that completed baseline. Participants reported that they were primarily male-identifying and heterosexual. When compared to the baseline cohort of 985, participants in this study were similar, except for a higher proportion who spoke English as their main language at home (91.2% in this study compared to 84.7% at baseline). Characteristics of the schools and students that participated in the trial are reported elsewhere (King *et al*. submitted). Participants in this study who provided responses to open-ended questions were from 11 secondary schools across two Australian states: New South Wales and Victoria. The majority were from urban, independent, all-boys schools in New South Wales. See Table 2 for school and student demographic characteristics.

**Table 2.** School and student characteristics.

	Schools	Students
Number of schools (total); number of participants (total)	11	183
Location of school		
Urban	8	166 (90.7)
Rural	3	17 (9.3)
State		
Victoria	3	17 (9.3)
New South Wales	8	166 (90.7)
Western Australia	0	0 (0)
School type		
State	6	33 (18)
Independent	5	150 (82)
Catholic	0	0 (0)
Consent method		
Opt-in	1	1 (0.5)
Opt-out	10	182 (99.5)
Education type		
All-boys	3	136 (74.3)
Co-education	8	47 (25.7)
Total number of participants		**183**
Age [mean (SD)]		15.18 (0.50)
Age in years (range)		14–17
Gender (*n*, %)		
Male		178 (97.3)
Transgender male		1 (0.5)
Nonbinary/gender diverse		2 (1.1)
Another gender		1 (0.5)
Do not know		0 (0)
Prefer not to say		1 (0.5)
Sexuality (*n*, %)		
Straight or heterosexual		164 (89.6)
Gay or homosexual		4 (2.2)
Bisexual		6 (3.3)
Another sexuality		2 (1.1)
Do not know		4 (2.2)
Prefer not to say		3 (1.6)
English main language spoken at home (*n*, %)		167 (91.2)
Indigenous status (*n*, %)		
Neither Aboriginal nor Torres Strait Islander		179 (97.8)
Aboriginal		3 (1.6)
Torres Strait Islander		0 (0)
Both Aboriginal and Torres Strait Islander		1 (0.5)

SD, standard deviation; range (minimum–maximum); (*n*, %), count and column percentage.

Survey responses ranged from one-word answers to a few sentences per question. Participants were overwhelmingly positive about their experience within the workshop with almost all reporting that they ‘*loved every part*’ of the workshop and that there was ‘*nothing*’ that they disliked. However, not all participants had the same experience; some raised challenges, and the perceived impacts following the workshop varied. Three overarching themes were generated from analysis of the survey responses: (i) ‘Normalizing struggle and strengthening connection within the workshop’, (ii) ‘Defining the ‘Man Code’ within and postworkshop’, and (ii) ‘Talking openly with friends and family postworkshop’. Subthemes are also identified throughout as shown in Table 3.

**Table 3.** Summary of themes.

Theme	Subtheme
1. Normalizing struggle and strengthening connection within the workshop.	a. Safe space for self-expression.
	b. Witnessing peer vulnerability and struggle.
2. Defining the ‘man code’ within and postworkshop.	
3. Talking openly with friends and family postworkshop.	a. Understanding, trust and communication skills with friends.
	b. Finding it easier to talk to family.

### Normalizing struggle and strengthening connection within the workshop

Most participants perceived that the workshop created an environment where they could openly express vulnerabilities and difficulties without fear of judgement or alienation from others. As Student 171 expressed, ‘*I liked being in an open safe environment with people who understood what I’d been going through*’. Participants described that prior to the workshop, they were unaware that their peers experienced personal struggles. They reported that the opportunity to both express themselves and witness others disclose their difficulties, normalized the experience of having personal problems among peers, and created a sense of intimacy, inclusivity, connection, and community.

We developed two subthemes that describe what participants perceived as contributing to normalizing struggle and strengthening connection within the workshop: (i) ‘Safe space for self-expression’ and (ii) ‘Witnessing peer vulnerability and struggle’. These subthemes include elements of the workshop that participants characterized as facilitating safe self-expression and a deeper understanding of others, alongside aspects that some boys found challenging.

#### Safe space for self-expression

Many participants reflected that the facilitators played a key role in creating a welcoming, fun and engaging environment. They appreciated the facilitators’ relatability and how they cultivated an atmosphere of openness, connection, and perceived informality. Facilitators were described as funny, engaging, down to earth, relatable, nice, and chill. ‘*They [the facilitators] made us feel that we were safe and free to talk about anything*’ (Student, 62).

Participants frequently described feeling safe and understood within the workshop, finding it easy to share their opinions and experiences in a secure environment. Student 83 noted, ‘I *liked the security and safety that was felt inside the workshop*’. Student 54 added, ‘*I was able to express my inner thoughts in a way that made me feel like I would not be judged*’. Many participants enjoyed discussing personal experiences, including mental health, while feeling that others listened attentively. Student 178 reflected, ‘*I liked the support you got, and that people sat there and listened [and] didn’t butt in*’. Some described a sense of alignment or common ground with their peers, due to being able to ‘*share our deepest memories with each other and be on the same page*’ (Student 118). This experience of feeling safe, heard, and understood during the workshop appeared to bridge the gap between authentic experiences and perceived social acceptance, offering boys an opportunity to feel validated rather than experiencing shame or stigma regarding vulnerability.

Although almost all participants reported positive experiences, some described aspects of the workshop that they did not like. Within these aspects, a common sentiment was feeling ‘*awkward*’. Student 66 expressed, ‘*The attitude going in is like it's a very safe environment and no one is forcing you to say anything, but they are at the same time. Also, teachers are there so you don't feel safe to speak with this random stranger*’. Student 127 added, ‘*It set expectations for kids to leave a comfortable zone and repeated the same stuff that is always told to us*’. These perspectives reflect feeling pressured to share personal stories with unfamiliar peers and facilitators, and a limited sense of safety due to the presence of teachers. These sentiments also indicate a frustration with a familiar onus on young people to be vulnerable. A couple of participants noted that they did not like anything about the workshop and one reported that they did not want to be there but did not provide any further explanation. Notably, boys that reported feeling awkward, also discussed enjoying the sharing as a group and feeling that the environment was welcoming overall. These sentiments suggest that boys may have been able to tolerate and arguably saw value in the discomfort associated with vulnerability when it occurred in a supportive environment. Nonetheless, that a few participants expressed criticism warrants attention, as others with similar feelings might not have completed the survey at all, meaning that their perspectives may not be fully represented in our sample.

#### Witnessing peer vulnerability and struggle

Participants discussed witnessing their peers open up about personal difficulties. They described this as a pivotal experience, impacting their understanding of both their own struggles and those of others, while strengthening their sense of connection as a group. Student 37 explained, ‘*I liked getting an understanding that everyone around you has issues; that you’re not the only one*’. Student 78 added. ‘*I didn’t feel alone anymore. The session really bonded the group*’. These reflections indicate that participants were previously unaware that their peers faced difficulties similar to themselves, and that the workshop illuminated having challenges as a shared human experience. Some participants described an active internal process of reflecting on how the experiences of others mirrored their own. Student 175 noted, ‘*I liked hearing other people’s problems and relating them to my life*’ (Student 175). This newfound awareness appeared to not only normalize vulnerability and struggle among participants but offer relief from feelings of isolation, cultivating what participants described as ‘*intimacy*’ and ‘*community*’.

The ‘Step to the Line’ activity was highlighted by many as impactful due to allowing nonverbal disclosure of personal information among peers. Participants noted that stepping to the line if a statement was true, such as having cried recently, enabled deeper self-expression, increased understanding of others, and lessened feelings of isolation. Student 83 observed, ‘*Everyone opened up and didn’t alienate you if you had an issue, especially in the ‘Step to The Line’ activity*’, indicating that the activity cultivated an environment where vulnerability was accepted rather than stigmatized. Other participants explained, ‘*People can open up about darker topics without the use of words*’ (Student 84) and ‘*…everyone could be honest about important issues without having to speak or justify themselves*’ (Student 120). These sentiments reflect that many participants valued the opportunity to disclose sensitive information among peers without the added challenge of verbal articulation.

Many participants indicated that the participant-centred, experiential style of the workshop encouraged agency, and ‘*progressive*’ modelling of vulnerability among peers (i.e. a gradual increase in talking openly within the group). As Student 50 described, ‘*I liked that the presenter waited for someone to go up and talk about their problems and that one person made everyone else feel comfortable to speak up*’. This feedback illustrates how the workshop’s design and facilitation were perceived as creating an environment where participants could practice acting outside of dominant norms. By allowing vulnerability to emerge within the group, the workshop leveraged the impact of peer modelling to create safety and inspire others to similarly engage. One participant, however, expressed concern about the possibility of being chosen to share past upsetting experiences, stating ‘*I did not like not knowing if we were going to be picked to open-up about past traumas*’ (Student 85). This apprehension highlights the delicate balance required in creating spaces for vulnerability while respecting participants’ autonomy and boundaries, particularly around sensitive experiences. Despite these concerns, participants generally found value in the workshop’s challenging nature, with Student 72 noting that the workshop ‘*was confronting but still enjoyable*’.

### Defining the ‘Man Code’ within and postworkshop

Participants reported varied impacts on their desired masculine identities. Many indicated that the workshop changed their ideas about the kind of man that they aspire to be and articulated a desire to increase their emotional expression and cultivate more supportive relationships. These reported shifts in perception involved challenging traditional masculine stereotypes (the ‘man code’) and developing a greater understanding of men’s mental health struggles. Some participants noted that they ‘*liked the open discussion about issues men have with mental health [and] talking about what society expects of us*’ (Student, 36).

Participants frequently reflected on a shift towards viewing vulnerability as ‘*brave*’ rather than weak and questioning social expectations of ‘*strength*’ as a defining characteristic of masculinity. Student 62 noted, ‘*[The workshop] showed me that I don’t always have to be the strong one*’. Student 111 added, ‘*The workshop changed my idea that men must keep their feelings bottled up*’. One participant used the metaphor of a ‘trap’ to describe the restrictive norms or ‘code’ that society has for men, suggesting a perceived newfound awareness of the potential harms of some masculine norms and how to resist them: ‘*[The workshop] changed my ideas about the man code and how not to fall into the trap of it*’ (Student 112).

Many participants discussed how their changed views of vulnerability and self-expression impacted their future relational aspirations. They reported wanting to behave ‘*outside of the man code*’, embrace open expression, and become better role models. This included feeling more confident and comfortable with emotion and noting that open expression does not diminish masculinity. As Student 10 expressed, ‘*It changed the way that I am allowed to express myself—in any way shape or form—and it doesn’t make me less of a man to anyone*’. Student 174 added, ‘*[The workshop] showed me that men can be closer and allow each other to be more sensitive together*’. Many participants discussed wanting to create mutually supportive and empathetic relationships, including ‘*wanting to be more open to listening to others*’ (Student 128) and to be ‘*more kind*’. Student 26 noted, ‘*I want to be someone that is there for the people I care about*’. One participant discussed wanting to improve generational cycles of modelled masculinity: ‘*It made me want to be a better man or father figure for my child than I had*’ (Student, 178).

Conversely, a large group of participants reported little or no change in their views of masculinity. Many of these participants did not provide further explanation. Many others attributed this lack of change in their views of masculinity to pre-existing beliefs consistent with the workshop’s focus on open emotional expression and communication for men, describing that they have never felt connected to the ‘man code’. Some discussed how the workshop’s ideas aligned with their existing values, such as recognizing the coexistence of interdependence, leadership, and strength. As Student 73 explained, ‘*I always thought that I would become a person who was strong but also relied on their partner and those around them, because that's what I think a strong leader is, and that is what I want to be*’.

Some participants expressed disagreement with the messages of the workshop and not liking talking about what it means to be a man. Student 16 reported that ‘*men are men for a reason… and society won’t change immediately even if we do*’, potentially reflecting an adherence to traditional masculine norms and a defence against evolving masculinities. Moreover, this perspective highlights how perceived futility of individual change against broader structural constraints may limit the workshop’s impact for some boys. A few participants also noted the impact of the workshop on their ideas of what sort of man they would like to be wearing off over time. ‘*In the moment it changed my thoughts [about what sort of man I would like to be] but after a little bit I forgot what we talked about*’ (Student 77).

### Talking openly with friends and family following the workshop

Participants reported varied impacts on talking openly with friends and family following the workshop. Two subthemes were generated as best explaining the factors contributing to talking openly with friends and family: (i) ‘Understanding, trust and communication skills with friends’, and (ii) ‘Finding it easier to talk to family’.

#### Understanding, trust and communication skills with friends

Many participants described talking more openly and deeply with their mates following the workshop and having increased trust that others would listen. For these participants, the workshop appeared to create a foundation for more authentic relationships, characterized by a shift towards more personal and meaningful dialogue. Student 52 noted, ‘*We talk more openly about our issues now instead of just being quiet about it*’. Participants discussed this openness as being underpinned by enhanced mutual trust. *[After the workshop] I know, and they know, we can trust each other more*’ (Student, 171). These reflections indicate increased relational security that extended beyond general openness to more vulnerable personal matters. As Student 158 reported, ‘*[The workshop] helped me know that I can talk with my mates about mine and their personal things*’. Student 84 added, ‘*[We] have started talking about our personal lives*’. A few participants also discussed becoming informal sources of mental health support for their peers. ‘*My mates now come to me for mental help, and I am happy to listen and help*’ (Student 85).

Participants frequently reported enhanced understanding and empathy towards their friends. Some participants discussed having increased communication skills, such as knowing how to initiate deeper conversations, asking how others are more often, and using open-ended questions when talking to their friends. As Student 162 reported, ‘*I don’t ask yes or no questions now when asking how someone is doing*’. Student 94 added, ‘*It did change the way I talk to my mates. I try to be more sensitive and understanding, figuring out when to stop talking and when to start talking*’. These responses indicate a perceived developing awareness of social-emotional cues that includes conversational reciprocity, tuning into the needs of others, holding space, and providing input thoughtfully. Some participants discussed applying this increased social-emotional awareness to traditionally masculine forms of interaction, namely banter. Student 122 reflected ‘*I would say that I am more compassionate talking to my friends now and I am more careful about what I say when I banter with them*’.

Others reported changing the way that they talk to their mates ‘a little bit’. These participants described being ‘a bit’ more open about what is going on in their lives and ‘*acting closer together*’, even if they did not change the way they talk. Some discussed that although they had not yet felt the need, they believed that they would be able to talk about personal problems with their friends if required. However, others discussed social barriers to attempts to discuss personal problems with friends, such as attempts not being reciprocated or what they shared not being believed. As Student 69 disclosed, ‘*I try to talk more openly but they [my friends] don’t*’. Some participants also said that the change in the way they talk to their peers ‘*wears off*’ and suggested it would be helpful if conversations continued in mentor groups or that workshops ran once a term to provoke sustained change.

Many participants also reported that the workshop did not change how they talk to their friends. The majority of these did not provide any further explanation. However, a large group attributed this lack of change to their pre-existing open communication with friends, reporting that they ‘*have always tried to be supportive and ask deeper questions*’ (Student 98) and ‘*have always let my mates know about my personal life*’ (Student, 31). Such sentiments reflect a perception among these participants that their friendships were already characterized by empathy and openness, as encouraged by the workshop. As Student 157 stated, ‘*I always talk to my mates with kindness and let them know I’m here for them*’.

#### Finding it easier to talk to family

Many participants discussed ‘*feeling more appreciative*’ of family and ‘*loved ones*’ following the workshop and ‘*finding it easier*’ to talk to family about ‘*personal stuff*’. These reflections indicate that the workshop enhanced many participants’ recognition of family as a source of support and connection and encouraged communication of personal problems within family networks. Student 94 commented, ‘*I release more information than I used to [to my family] and find it better to communicate*’. Some participants reported ‘*having a long talk*’ and conversations about ‘*being a man*’ with their family following the workshop, creating an opportunity for broader family-level reflection regarding masculine norms. Some highlighted a change in how they communicate with particular family members, such as their mother or with their parents (but not siblings). As Student 20 reflected, ‘*I am now more keen to talk to my mum about personal stuff, compared to before, I would just lock myself in my room*’.

Conversely, many participants reported that the workshop did not change how they talked with their family. The majority of these did not provide any further explanation. A large group, however, attributed the lack of change to pre-existing open communication with their family. Student 73 noted, ‘*I’ve always been very open with my family*’. A few discussed that they ‘*already talk best with my family*’ (Student 164). Alternatively, a few participants noted that they are least likely to talk openly to their family. As Student 178 reported, ‘*I don’t open-up, especially to them [my family]*’. These responses highlight the importance of pre-existing family dynamics that may either facilitate or complicate the workshop’s impact within each participants’ unique family network.

Most participants believed that their family would be supportive, caring and try to help them, including seeking professional mental health support, if needed. However, a large minority reported that their family would not respond well, which included a lack of response, not taking it seriously or that their family would ‘*try to help but overall become too involved and make it embarrassing and annoying*’ (Student, 134). Others expressed that their family would have an emotional reaction, which included being sad, worried, anxious, or shocked. A few reported that they thought their parents would reinforce traditional masculine norms such as telling them to be strong or thinking that they are weak. A few others acknowledged their families’ desire to help them, however, still did not want to let them know if they were upset.

## DISCUSSION

This study explored participant experiences of the ‘Breaking the Man Code’ workshop and its perceived impact on help-seeking about personal problems with friends and family. Participants were 183 adolescent boys from the intervention arm of a trial (King *et al*. submitted). Our sample comprised just over half of all participants in the intervention follow-up group of the trial. Participants in this study were largely heterosexual and male-identifying, with an overrepresentation of urban, all-boys schools in New South Wales. Most participants described that the workshop created a safe environment for self-expression, where they realized that their peers faced difficulties similar to themselves, which strengthened perceived peer connections. Almost all participants reported highly positive experiences within the workshop, while some noted challenges. Reported change following the workshop was heterogeneous. Many participants reported redefining their views on the type of man that they would like to be, reframing vulnerability as bravery, and expressing a desire for increased self-expression and support for others. Likewise, many participants perceived a shift in their behaviour, noting that they talked more openly with friends and family about personal problems. Many participants also reported no changes. Of these, most attributed the lack of change to pre-existing alignment with the workshop’s ideas and established open communication practices with their friends and family. Other participants that reported a lack of change did not provide an explanation, while some reported difficulties with implementing and sustaining change.

Our results indicate a perceived impact for many boys in this sample that accord with the programme’s aims of disrupting rigid masculine norms, fostering a more expressive version of manhood, building healthier connections, and increasing the ability to engage in meaningful conversations (https://www.tomorrowman.com.au/education-programs/student-program). Our findings add valuable nuance to the results of the broader trial from which these participant responses were drawn from King *et al*. (submitted). The qualitative findings reported here are consistent with the trial’s closed ended questions, which indicated increased help-offering and connection to friends. However, our findings diverge from those related to the trial’s primary outcome measure, the General Help-Seeking Questionnaire (GHSQ) (Wilson *et al*. 2005), which found no significant change in mean help-seeking intentions (King *et al*. submitted). Further investigation is needed to better understand differential impacts. The qualitative findings of the current study provide valuable insights into subtle shifts in intentions, attitudes, and behaviours that quantitative measures, which focus on average changes across a population, may not fully capture. Our study highlights the importance of incorporating qualitative approaches to evaluate complex interventions, particularly those addressing multifaceted, socially embedded, constructs like masculine norms.

Our findings contribute to an emerging body of knowledge regarding gender transformative interventions. For example, Calear and colleagues’ (2021) evaluated the ‘Silence is Deadly’ programme, another upstream gender transformative school-based intervention in Australia. This programme utilizes role modelling, personal narratives, and psychoeducation, aiming to increase help-seeking among adolescent boys. The authors found a quantitative increase in help-seeking intentions from friends, and qualitative reports from school staff indicated positive changes in boys’ help-seeking behaviour. Our findings in the current study are consistent with this pattern: many boys reported perceived positive attitudinal and behavioural impacts. These converging findings offer promising results of school-based gender transformative interventions aimed at promoting help-seeking among adolescent boys.

Our results highlight key elements of the workshop that many participants perceived as creating a safe space for open expression: empathetic and relatable facilitators, an experiential design that enabled peer leadership (e.g. by waiting for one student to speak up and others then being able to follow suit), and the inclusion of nonverbal activities. These findings align with recommendations for an indirect approach to shifting adherence to traditional masculine norms rather than relying on didactic methods (Kiselica and Englar-Carlson 2010, Wilson *et al*. 2022) and to create environments that prioritize equitable power dynamics, genuine care, and respect to encourage help-seeking among young men (Lynch *et al*. 2018). These elements highlighted by participants expand on previous research indicating positive impacts of gender transformative programmes that incorporate role modelling, personal narratives, and psychoeducation (Calear *et al*. 2021) and offer pathways for future research aimed at understanding impactful elements of gender transformative interventions for adolescent boys (Pirkis *et al*. 2019). The positive workshop experiences reported by participants suggest that many adolescent boys in our sample were receptive to and valued, safe and supported gender transformative approaches, even when challenging or ‘*awkward*’. The potential for concern among some participants about being selected to share difficult past experiences with their peers, highlights the importance of participant-led approaches that centre the autonomy of adolescent boys regarding whether they speak in front of the group and how much personal information they decide to share during the workshop. Future research should continue to explore perspectives on the workshop elements highlighted by participants in this study with a more diverse group, such as increased representation of rural and state schools, to inform strengths-based approaches and to enhance standardization, construct validity, and ongoing rigorous evaluation (Flake *et al*. 2017).

The finding that many participants redefined their masculinities to include increased self-expression and a desire to create more intimate and supportive relationships echoes the qualitative results of King and colleagues (2018) ‘Man Up’ evaluation (a multimedia suicide prevention intervention for men), where adult men reported developing greater awareness of the personal problems of other men, and an increased desire to offer support. This aligns with research emphasizing the importance of cultivating mutually supportive friendships (Smiler 2014) and shared experiences (i.e. safe open expression during the workshop) (Burke *et al*. 2022) when encouraging emotional expressiveness and disclosure of personal problems among adolescent boys. The description of masculinity described by participants aligns with Wilson and colleagues (2022) school-based framework of positive masculinity, which emphasizes connection, self-motivation, and authenticity. These attributes are juxtaposed with traits such as self-reliance, thwarted social support and a reluctance to seek help, which are linked to increased risk of suicide in boys and men (Mergl *et al*. 2015; Player *et al*. 2015).

The perceived increase in open communication with friends and family reported by many participants following the workshop suggests that gender transformative interventions can facilitate behavioural shifts in adolescent boys that may reflect changes in adherence to traditional masculine norms. Participants’ more detailed description of impacts with friends—including increased social-emotional awareness, trust, connection, compassion, and use of active listening skills—highlight relational changes conducive to help-seeking by creating room for vulnerability (Rickwood *et al*. 2005, Seidler *et al*. 2016, Rasmussen *et al*. 2018). The reported application of active listening skills with friends supports recommendations for gender transformative interventions to equip boys with practical skills to translate intentions and attitudes into behaviour change, and overcome initial awkwardness (Elliott *et al*. 2022). Future research should investigate the long-term impact of these interventions on communication, exploring participants’ ongoing use of active listening skills, whether the use of these skills extends to friends that have not participated in a workshop, relationships beyond friends, and what factors support or hinder the continued use of skills.

Given that some participants noted difficulties, such as friends not reciprocating attempts at open communication, further research is needed to explore whether unmatched increased desire for open expression among friends has negative impacts such as feeling isolated or motivates seeking alternative friendships following the workshop (Smiler 2014). Participant perspectives of the current study also align with findings from Elliott and colleagues (2022) evaluation of the ‘Realising My Potential’ workshop, an upstream gender transformative intervention for boys (aged 12–16 years) that aims to prevent suicide, depression, and gender-based violence. Focus group data from the authors’ evaluation found that participants perceived changes in how they talk to friends diminishing over time. In accord with our findings, they recommend ongoing practice groups led by a trained facilitator to sustain changes. While Tomorrow Man offers a long-form programme, a cost-effective alternative may be to upskill school staff to facilitate ongoing group activities. Noting our finding that teachers’ presence can limit the perceived safety for some students, alternatives could include ongoing trained peer-led or youth mentor groups.

Our findings suggest the potential of gender transformative interventions to increase open communication of personal problems not only with peers but also within families. The reported variations in changes with family members reaffirm the need to continue to consider adolescent boys’ unique family dynamics when implementing and understanding impacts of school-based interventions. Given that low perceived family support is linked to low general help-seeking intentions among Australian adolescent males (Liddle *et al*. 2021) and anticipating burden is a known barrier to help-seeking from family among adolescents (Gulliver *et al*. 2010), future research should consider the influence of these factors on workshop impacts. Recognizing the need for community-wide support, Tomorrow Man offers workshops for parents and teachers alongside those for students. Future research should investigate the impact of involving multiple agents of change, such as through parent-child dyad research where both have attended a workshop, or school case studies where student, parent, and teacher workshops have occurred, to better understand the influence on familial relational dynamics and community-level impact.

This study highlights heterogeneity in the impacts of gender transformative workshops for adolescent boys, indicating the need for a more nuanced understanding of adolescent masculinities. While many participants reported positive changes, the lack of perceived change reported by others may indicate that some boys already possess flexible masculine identities and open communication practices, aligning with the view that contemporary adolescent masculinities are becoming more diverse (Anderson and McCormack 2018). A recent qualitative case study exploring conceptualizations of masculinities within an urban all-boys private school in Melbourne, Australia, found promising results, indicating minimal adherence to inflexible self-reliance norms and greater willingness to express emotions among adolescents within the case-school (O’Gorman *et al*. 2025). Conversely, the lack of perceived change reported in this study may also be due to broader social pressures that made enacting behavioural change too difficult, as indicated by some participants in our sample and in the broader trial results (King *et al*. submitted). Given the varied perspectives and impacts reported in the current study, investigating the presence and influence of varying masculine norm conformity profiles among adolescent boys, similar to those identified in adult men (Eggenberger *et al*. 2024), may be valuable. This could inform the development of more tailored programmes, as those adhering more strongly to restrictive norms may experience greater social barriers to change.

As we were unable to ask follow-up questions among participants, future research utilizing qualitative interviews or focus groups with adolescent boys is needed to better understand differing impacts. Some boys that reported no change in their direct communication nonetheless discussed subtle shifts in peer relationships, including increased closeness and a sense that they could talk openly if needed. These findings may warrant consideration of how upstream interventions are evaluated. For example, evaluations that rely on quantitative measures of help-seeking or focus only on behaviour change may miss subtle impacts in peer relational quality, connection, or perceived safety. Future research could consider the use of evaluation methods that are better equipped to explore impacts on social bonds and intersections with shame, stigma, distress and suicidality (Franklin *et al*. 2018). Other researchers have similarly called for greater emphasis on the interpersonal context of help-seeking in suicide prevention efforts aimed at encouraging open communication among boys and men (Chandler 2022).

This study’s strengths include a real-world setting and an open-ended anonymous survey design, conducive to eliciting candid responses on sensitive topics from adolescent boys and to gather rich qualitative data from large, geographically diverse populations, suitable for emerging research topics (Edwards and Holland 2020, Braun *et al*. 2021, Thomas *et al*. 2024). Methodological limitations, however, warrant consideration. First, the overrepresentation of heterosexual male-identifying participants from urban, independent, all-boys schools limits the generalizability of our findings. Future research should include more diverse samples, particularly considering boys from rural and state schools, and intersecting populations at heightened risk of suicide including Aboriginal and Torres Strait Islander youth and the need for interventions to be culturally informed (Sheikh *et al*. 2025). Second, the length of the questionnaire battery may have introduced a self-selection bias, potentially favouring motivated participants. Third, it’s possible that social desirability may have influenced participants who completed the survey together with their peers, within school settings. However, it is unknown what impact this may have had on the responses in this study. Finally, albeit an important perspective, our study relies on participant interpretation. Future studies should explore additional key perspectives such as parents, school, and Tomorrow Man staff to further understand impacts. Additionally, understanding the influence of broader barriers to engagement, such as the ‘manosphere’—an online network of interest groups and influencers that aim to reinforce the patriarchy (Ging 2019, Wescott *et al*. 2024)—will be crucial for developing comprehensive and effective interventions.

## CONCLUSION

This study contributes to emerging knowledge regarding school-based gender transformative interventions aimed at reframing rigid masculine norms and increasing flexible self-expression, including the discussion of personal problems (i.e. help-seeking) among adolescent boys. By shifting masculine norms towards vulnerability and help-seeking, such programmes may have upstream suicide preventative impacts within the community. Given that men represent almost 75% of suicide deaths in Australia, evidence illuminating the impacts of this upstream gender transformative programme addresses a critical need. Participants were generally positive about their experience within the workshop. Many boys in this study indicated that they possessed both the desire and the capacity to develop a positive masculinity characterized by deeper social connections, reporting perceived progress towards this shift following the workshop. However, many also reported no changes postworkshop and others reported challenges sustaining new behaviours. The findings of this study may inform further refinement of the programme. Future research is needed to better understand varied impacts among diverse boys. Ongoing community-wide approaches are required to sustain positive changes.

## Data Availability

The data underlying this article cannot be shared publicly due to protection of the privacy of schools and individuals that participated in the study.

## References

[daaf160-B1] Anderson E, McCormack M. Inclusive masculinity theory: overview, reflection and refinement. J Gend Stud 2018;27:547–61. 10.1080/09589236.2016.1245605

[daaf160-B2] Australian Bureau of Statistics . *Causes of Death, Australia*. 2023. https://www.abs.gov.au/statistics/health/causes-death/causes-death-australia/2022 (12 May 2025, date last accessed).

[daaf160-B3] Australian Institute of Health and Welfare . *Suicide and Intentional Self-harm Hospitalisations Among Young People.* 2025. https://www.aihw.gov.au/suicide-self-harm-monitoring/population-groups/young-people/suicide-self-harm-young-people (5 August 2025, date last accessed).

[daaf160-B4] Braun V, Clarke V. Using thematic analysis in psychology. Qual Res Psychol 2006;3:77–101. 10.1191/1478088706qp063oa

[daaf160-B5] Braun V, Clarke V. Thematic Analysis: A Practical Guide. London: SAGE Publications Ltd, 2022.

[daaf160-B6] Braun V, Clarke V, Boulton E et al The online survey as a qualitative research tool. Int J Soc Res Methodol 2021;24:641–54. 10.1080/13645579.2020.1805550

[daaf160-B7] Burke L, John M, Hanna P. A qualitative exploration of how young men in the UK perceive and experience informal help-seeking for mental health difficulties. Child Youth Serv Rev 2022;137:106440. 10.1016/j.childyouth.2022.106440

[daaf160-B8] Calear AL, Morse AR, Batterham PJ et al Silence is Deadly: a controlled trial of a public health intervention to promote help-seeking in adolescent males. Suicide Life Threat Behav 2021;51:274–88. 10.1186/s12889-017-4845-z 33876483

[daaf160-B9] Canetto S, Sakinofsky I. The gender paradox in suicide. Suicide Life Threat Behav 1998;28:1–23. 10.1111/j.1943-278X.1998.tb00622.x 9560163

[daaf160-B10] Chandler A . Masculinities and suicide: unsettling ‘talk’ as a response to suicide in men. Crit Public Health 2022;32:499–508. 10.1080/09581596.2021.1908959

[daaf160-B11] Chu JY, Porche MV, Tolman DL. The adolescent masculinity ideology in relationships scale: development and validation of a new measure for boys. Men Masc 2005;8:93–115. 10.1177/1097184X03257453

[daaf160-B12] Clark LH, Hudson JL, Rapee RM et al Investigating the impact of masculinity on the relationship between anxiety specific mental health literacy and mental health help-seeking in adolescent males. J Anxiety Disord 2020;76:102292. 10.1016/j.janxdis.2020.102292 33010663

[daaf160-B13] Cleary A . Suicidal action, emotional expression, and the performance of masculinities. Soc Sci Med 2012;74:498–505. 10.1016/j.socscimed.2011.08.002 21930333

[daaf160-B14] Cole BP, Ingram PB. Where do I turn for help? Gender role conflict, self-stigma, and college men’s help-seeking for depression. Psychol Men Masc 2020;21:441–52. 10.1037/men0000245

[daaf160-B15] Connell RW . Growing up masculine: rethinking the significance of adolescence in the making of masculinities. Ir J Sociol 2005;14:11–28. 10.1177/079160350501400202

[daaf160-B16] Di Bianca M, Mahalik JR. A relational-cultural framework for promoting healthy masculinities. Am Psychol 2022;77:321–32. 10.1037/amp0000929 35587398

[daaf160-B17] Edwards R, Holland J. Reviewing challenges and the future for qualitative interviewing. Int J Soc Res Methodol 2020;23:581–92. 10.1080/13645579.2020.1766767

[daaf160-B18] Eggenberger L, Spangenberg L, Genuchi MC et al Men’s suicidal thoughts and behaviors and conformity to masculine norms: a person-centered, latent profile approach. Heliyon 2024;10:e39094. 10.1016/j.heliyon.2024.e39094 39640782 PMC11620066

[daaf160-B19] Elliott R, Fischer CT, Rennie DL. Evolving guidelines for publication of qualitative research studies in psychology and related fields. Br J Clin Psychol 1999;38:215–29. 10.1348/014466599162782 10532145

[daaf160-B20] Elliott K, Roberts S, Ralph B et al Evaluating Programs Aimed at Gender Transformative Work with Men and Boys: A Multicohort, Cross-Sector Investigation. Melbourne: The Victorian Health Promotion Foundation, 2022.

[daaf160-B21] Flake JK, Pek J, Hehman E. Construct validation in social and personality research: current practice and recommendations. Soc Psychol Personal Sci 2017;8:370–8. 10.1177/1948550617693063

[daaf160-B22] Flood M, The Men’s Project. Unpacking the Man Box: What Is the Impact of the Man Box Attitudes on Young Australian Men’s Behaviours and Wellbeing? Melbourne: Jesuit Social Services, 2020. https://jss.org.au/programs/research/unpacking-the-man-box-report/(11 August 2025, date last accessed)

[daaf160-B23] Franklin A, Barbosa Neves B, Hookway N et al Towards an understanding of loneliness among Australian men: gender cultures, embodied expression and the social bases of belonging. J Sociol 2018;55:124–43. 10.1177/1440783318777309.

[daaf160-B24] Galambos NL, Almeida DM, Petersen AC. Masculinity, femininity, and sex role attitudes in early adolescence: exploring gender intensification. Child Dev 1990;61:1905–14. 10.2307/1130846 2083504

[daaf160-B25] Ging D . Alphas, betas, and incels: theorizing the masculinities of the manosphere. Men Masc 2019;22:638–57. 10.1177/1097184X17706401

[daaf160-B26] Gulliver A, Griffiths KM, Christensen H. Perceived barriers and facilitators to mental health help-seeking in young people: a systematic review. BMC Psychiatry 2010;10:113. 10.1186/1471-244X-10-113 21192795 PMC3022639

[daaf160-B27] Gwyther K, Swann R, Casey K et al Developing young men’s wellbeing through community and school-based programs: a systematic review. PLoS One 2019;14:e0216955. 10.1371/journal.pone.0216955 31107908 PMC6527294

[daaf160-B28] Herreen D, Rice S, Currier D et al Associations between conformity to masculine norms and depression: age effects from a population study of Australian men. BMC Psychol 2021;9:1–10. 10.1186/s40359-021-00533-6 33608063 PMC7893732

[daaf160-B29] Iwamoto DK, Brady J, Kaya A et al Masculinity and depression: a longitudinal investigation of multidimensional masculine norms among college men. Am J Mens Health 2018;12:1873–81. 10.1177/1557988318785549 29973104 PMC6199432

[daaf160-B30] Jackson EF, Bussey K, Myers E. Encouraging gender conformity or sanctioning nonconformity? Felt pressure from parents, peers, and the self. J Youth Adolesc 2021;50:613–27. 10.1007/s10964-020-01387-8 33442774

[daaf160-B31] King K, Clark A, Trevena J et al Cluster randomised trial of the impact of the *Breaking the Man Code* workshops on adolescent boys’ intentions to seek help. (submitted). 10.1186/s13063-022-06034-0 PMC8811738 35115023

[daaf160-B32] King K, Schlichthorst M, Chondros P et al Protocol for a cluster randomized control trial of the impact of the Breaking the Man Code workshops on adolescent boys’ intentions to seek help. Trials 2022;23:110. 10.1186/s13063-022-06034-0 35115023 PMC8811738

[daaf160-B33] King K, Schlichthorst M, Keogh L et al Can watching a television documentary change the way men view masculinity? J Men’s Stud 2018;27:287–306. 10.1177/1060826518815909.

[daaf160-B34] King TL, Shields M, Sojo V et al Expressions of masculinity and associations with suicidal ideation among young males. BMC Psychiatry 2020;20:228. 10.1186/s12888-020-2475-y 32398056 PMC7218581

[daaf160-B35] Kiselica MS, Englar-Carlson M. Identifying, affirming, and building upon male strengths: the positive psychology/positive masculinity model of psychotherapy with boys and men. Psychotherapy (Chic). 2010;47:276–87. 10.1037/a0021159 22402085

[daaf160-B36] Levant RF, Wimer DJ, Williams CM et al The relationships between masculinity variables, health risk behaviors and attitudes toward seeking psychological help. Int J Men’s Health 2009;8:3–21. 10.3149/jmh.0801.3

[daaf160-B37] Liddle SK, Robinson L, Vella SA et al Profiles of mental health help seeking among Australian adolescent males. J Adolesc 2021;92:34–45. 10.1016/j.adolescence.2021.08.010 34416479

[daaf160-B38] Lynch L, Long M, Moorhead A. Young men, help-seeking, and mental health services: exploring barriers and solutions. Am J Men’s Health 2018;12:138–49. 10.1177/1557988315619469 27365212 PMC5734535

[daaf160-B39] Madill A, Jordan A, Shirley C. Objectivity and reliability in qualitative analysis: realist, contextualist and radical constructionist epistemologies. Brit J Psychol 2000;91:1–20. 10.1348/000712600161646 10717768

[daaf160-B40] Martino W, Meyenn B. What About the Boys? Issues of Masculinity in Schools. Philadelphia, PA: Open University Press, 2001.

[daaf160-B41] McCarthy S, Thomas SL, Pitt H et al “They loved gambling more than me.” Women’s experiences of gambling-related harm as an affected other. Health Promot J Austr 2023;34:284–93. 10.1002/hpja.608 35470511

[daaf160-B42] Mergl R, Koburger N, Heinrichs K et al What are reasons for the large gender differences in the lethality of suicidal acts? An epidemiological analysis in four European countries. PLoS One 2015;10:e0129062. 10.1371/journal.pone.0129062 26147965 PMC4492725

[daaf160-B43] Nielson MG, Martin CL, Rogers LO et al Exploring young men’s experience of resistance to masculine norms. Emerg Adulthood 2022;11:365–79. 10.1177/21676968211072933

[daaf160-B44] O’Gorman K, Fisher K, Wilson MJ et al “The Landscape’s Changed and No One’s Giving them a New Map”: positive masculinity in boys and young men. Int J Men’s Soc Community Health 2025;8:36–49. 10.3138/ijmsch.2024.0007

[daaf160-B45] Patton GC, Glover S, Bond L et al The gatehouse project: a systematic approach to mental health promotion in secondary schools. Aust N Z J Psychiatry 2000;34:586–93. 10.1080/j.1440-1614.2000.00718.x 10954389

[daaf160-B46] Pirkis J, Schlichthorst M, King K et al Looking for the ‘active ingredients’ in a men’s mental health promotion intervention. Adv Ment Health 2019;17:135–45. 10.1080/18387357.2018.1526095

[daaf160-B47] Player MJ, Proudfoot J, Fogarty A et al What interrupts suicide attempts in men: a qualitative study. PLoS One 2015;10:e0128180. 10.1371/journal.pone.0128180 26090794 PMC4474962

[daaf160-B48] Rasmussen M, Hjelmeland H, Dieserud G. Barriers toward help-seeking among young men prior to suicide. Death Stud 2018;42:96–103. 10.1080/07481187.2017.1328468 28489969

[daaf160-B49] Reigeluth CS, Addis ME. Adolescent boys’ experiences with policing of masculinity: forms, functions, and consequences. Psychol Men Masc 2016;17:74–83. 10.1037/a0039342

[daaf160-B50] Rice S, Purcell R, McGorry P. Adolescent and young adult male mental health: transforming system failures into proactive models of engagement. J Adolesc Health 2018a;62:S9–17. 10.1016/j.jadohealth.2017.07.024 29455724

[daaf160-B51] Rice SM, Telford NR, Rickwood DJ et al Young men’s access to community-based mental health care: qualitative analysis of barriers and facilitators. J Mental Health 2018b;27:59–65. 10.1080/09638237.2016.1276528 28132568

[daaf160-B52] Rickwood D, Deane FP, Wilson CJ et al Young people’s help-seeking for mental health problems. Austr E-J Adv Mental Health 2005;4:218–51. 10.5172/jamh.4.3.218

[daaf160-B53] Rickwood DJ, Deane FP, Wilson CJ. When and how do young people seek professional help for mental health problems? Med J Aust 2007;187:S35–9. 10.5694/j.1326-5377.2007.tb01334.x 17908023

[daaf160-B54] Rogers AA, Nielson MG, Santos CE. Manning up while growing up: a developmental-contextual perspective on masculine gender-role socialization in adolescence. Psychol Men Masc 2021;22:354–64. 10.1037/men0000296

[daaf160-B55] Rogers LO, Way N. “I Have Goals to Prove All Those People Wrong and Not Fit Into Any One of Those Boxes”: paths of resistance to stereotypes among black adolescent males. J Adolesc Res 2016;31:263–98. 10.1177/0743558415600071

[daaf160-B56] Seidler ZE, Dawes AJ, Rice SM et al The role of masculinity in men’s help-seeking for depression: a systematic review. Clin Psychol Rev 2016;49:106–18. 10.1016/j.cpr.2016.09.002 27664823

[daaf160-B57] Sheikh A, Payne-Cook C, Lisk S et al Why do young men not seek help for affective mental health issues? A systematic review of perceived barriers and facilitators among adolescent boys and young men. Eur Child Adolesc Psychiatry 2025;34:565–83. 10.1007/s00787-024-02520-9 39004687 PMC11868194

[daaf160-B58] Smiler AP . Resistance is futile? Examining boys who actively challenge masculinity. Psychol Men Masc 2014;15:256–9. 10.1037/a0037286

[daaf160-B59] Thomas SL, Pitt H, McCarthy S et al Methodological and practical guidance for designing and conducting online qualitative surveys in public health. Health Promot Int 2024;39:daae061. 10.1093/heapro/daae061 38920273 PMC11200187

[daaf160-B60] Wescott S, Roberts S, Zhao X. The problem of anti-feminist ‘manfluencer’ Andrew Tate in Australian schools: women teachers’ experiences of resurgent male supremacy. Gend Educ 2024;36:167–82. 10.1080/09540253.2023.2292622

[daaf160-B61] Wilson CJ, Deane FP, Ciarrochi J et al Measuring help seeking intentions: properties of the General Help Seeking Questionnaire. Can J Couns 2005;39:15–28. https://psycnet.apa.org/record/2005-05989-002

[daaf160-B62] Wilson M, Gwyther K, Swann R et al Operationalizing positive masculinity: a theoretical synthesis and school-based framework to engage boys and young men. Health Promot Int 2022;37:daab031. 10.1093/heapro/daab031 33842967

[daaf160-B63] World Health Organization . *Suicide Worldwide in 2019: Global Health Estimates*. Geneva. 2019. https://www.who.int/publications/i/item/9789240026643 (5 May 2025, date last accessed).

[daaf160-B64] World Health Organization . *Engaging Men and Boys in Changing Gender-Based Inequity in Health: Evidence from Programme*. Geneva. 2007. https://iris.who.int/handle/10665/43679 (5 May 2025, date last accessed).

[daaf160-B65] Yardley L . Dilemmas in qualitative health research. Psychol Health 2000;15:215–28. 10.1080/08870440008400302

